# Bridging In Vitro and Murine Breast Cancer Models: Advanced Imaging Across Multiscale Experimental Platforms

**DOI:** 10.3390/cancers18152469

**Published:** 2026-07-31

**Authors:** Cristina Terlizzi, Ylenia Ferrara, Annachiara Sarnella

**Affiliations:** Institute of Biostructure and Bioimaging, National Research Council, 80145 Napoli, Italy; cristina.terlizzi@ibb.cnr.it (C.T.); annachiara.sarnella@ibb.cnr.it (A.S.)

**Keywords:** breast cancer, multimodal imaging, organoids, patient-derived xenograft, preclinical models

## Abstract

Breast cancer is a highly heterogeneous disease characterized by distinct molecular subtypes, dynamic tumor–microenvironment interactions, and variable therapeutic responses. Despite the availability of multiple preclinical platforms, a major challenge remains the lack of a coherent multiscale framework capable of integrating biological complexity across experimental systems. This limitation reduces the predictive power of individual models and highlights the need for complementary strategies that reproduce disease progression across multiple biological scales. In this context, advanced imaging technologies have emerged as essential tools for linking preclinical platforms and enhancing their translational relevance. This review examines how multimodal imaging supports the integration of in vitro, ex vivo, and in vivo breast cancer models. We discuss how optical imaging, high-frequency ultrasound, magnetic resonance imaging, positron emission tomography/computed tomography, and intravital microscopy provide complementary molecular, functional, anatomical, and cellular information for the longitudinal assessment of tumor growth, metastatic dissemination, microenvironment remodeling, and therapeutic response. Particular attention is given to emerging translational workflows that combine patient-derived models with advanced imaging to investigate drug sensitivity, treatment resistance, and tumor progression within a precision oncology perspective. We also highlight the role of multimodal imaging in biomarker validation across platforms and in the development of clinically relevant preclinical pipelines. Overall, advanced imaging represents a critical translational bridge across breast cancer model systems, improving the predictive value of preclinical studies and supporting imaging-guided precision oncology from bench to bedside.

## Simple Summary

Breast cancer is a complex disease with significant differences among patients in terms of tumor features, disease progression, and response to treatment. Although several experimental models are available to study breast cancer, no single model can fully recapitulate the complexity of human tumors. This review highlights how the integration of complementary models, including patient-derived three-dimensional systems and animal models, with advanced imaging technologies, can provide a more comprehensive overview of tumor biology. Multimodal imaging enables the non-invasive and longitudinal monitoring of tumor growth, microenvironment changes, metastatic progression, and response to therapy, while supporting the identification of clinically relevant biomarkers. These integrated approaches can improve the predictive value of preclinical studies and contribute to the development of more personalized strategies for breast cancer diagnosis and treatment.

## 1. Introduction

Breast cancer (BC) remains the leading cause of cancer-related death among women worldwide, representing the second most frequently diagnosed malignancy and the fourth leading cause of cancer mortality overall [1]. Despite significant advances in early detection and therapeutic strategies, BC is a highly heterogeneous disease with a complex etiology driven by genetic susceptibility, environmental exposures, and lifestyle-related factors [2]. These drivers contribute to distinct geographic patterns of incidence, molecular subtypes, and clinical behavior. Moreover, aggressive BC subtypes continue to pose major therapeutic challenges due to high metastatic potential, treatment resistance, and poor prognosis, significantly impacting patient survival and quality of life. BC comprises distinct molecular subtypes with specific biological and clinical features, primarily classified as luminal, HER2-positive, and triple-negative breast cancer (TNBC). Luminal tumors are typically estrogen receptor (ER)- and/or progesterone receptor (PR)-positive, characterized by lower proliferative activity and a relatively favorable prognosis. HER2-positive cancers are characterized by HER2 amplification or overexpression, exhibiting variable aggressiveness and good responsiveness to targeted therapies. In contrast, TNBC lacks ER, PR, and HER2 expression, represents the most heterogeneous and aggressive subtype, and is frequently associated with high metastatic potential, therapeutic resistance, and poor clinical outcomes [2]. Notably, its aggressive behavior is also shaped by tumor microenvironmental factors, including stromal cell recruitment and interactions [3]. This molecular heterogeneity is a key driver of treatment response, disease progression, and therapeutic resistance. Accordingly, a deeper understanding of BC molecular subtypes and their tumorigenic variability is essential to improve in vivo preclinical models, thereby enhancing translational relevance and clinical trial design. Therefore, preclinical experimental approaches, including in vitro, ex vivo, and in vivo models, are indispensable for recapitulating BC complexity and supporting translational therapeutic research. Rather than providing a conventional overview of BC models, this review focuses on the integration of advanced imaging technologies across complementary experimental platforms, from organoids and CAM models to murine systems, highlighting their potential to create a multiscale translational framework for BC research.

### 1.1. In Vitro and Ex Vivo Models

In vitro and ex vivo models represent essential tools for investigating BC biology under controlled experimental conditions. Among these, conventional immortalized BC cell lines remain the most widely used model systems due to their ease of culture, reproducibility, and cost-effectiveness. Over the past decades, they have contributed substantially to the understanding of oncogenic signaling pathways, cellular proliferation, metastatic behavior, and therapeutic responses across different BC subtypes. Nevertheless, traditional two-dimensional (2D) cell cultures present significant limitations, as they fail to fully reproduce the structural organization of tumors, intra-tumoral heterogeneity, and the complex interactions between cancer cells, stromal components, and the extracellular matrix (ECM). As a result, their ability to predict clinical responses is often limited. To address these limitations, three-dimensional (3D) culture systems have emerged as more physiologically relevant models. Accordingly, a variety of approaches have been developed to more faithfully reproduce tumorigenesis and metastatic progression. Among these, spheroids have been widely used because they represent an easily achievable model that mimics the metastatic cascade and cancer cell migration in 3D. Their 3D structure reproduces tumor micro-regions and early micro-metastases, enabling the quantitative analysis of tumor cell invasion through advanced automated imaging and image analysis approaches. Vinci et al. have described a 3D tumor spheroid invasiveness assay that offers a rapid and automatable method for assessing invasiveness, based on a standardized and highly reproducible procedure, in a 96-well plate format with one spheroid per well. They demonstrated that this assay provides a rapid and highly reproducible method for assessing tumor cell invasion in a semisolid matrix, making it particularly suitable for future in vitro drug screening and personalized medicine applications using patient-derived spheroids [4].

Organoid technology has gained considerable attention because of its capacity to preserve tissue architecture and cellular diversity. Early advances in the field were achieved using non-malignant mammary tissue. Jardé et al. demonstrated that activation of the Wnt and Neuregulin-/ErbB signaling pathways enabled the long-term expansion of hormone-responsive mammary organoids while maintaining key features of the mammary epithelium, including distinct basal and luminal compartments, functional steroid hormone receptors, and stem/progenitor cell populations capable of regenerating a complete mammary gland in vivo. Furthermore, these organoids provided a valuable platform for investigating mammary gland development, hormone signaling, and the molecular mechanisms involved in breast tumorigenesis [5]. Building on these advances, patient-derived organoids (PDOs) have emerged as powerful ex vivo models for BC research. PDOs are established by isolating tumor cells from patient-derived specimens and culturing them within 3D ECM scaffolds supplemented with specific growth factors. Under these conditions, tumor cells self-organize into structures that partially recapitulate the architecture, cellular composition, and functional properties of the original tumor. Importantly, BC PDOs preserve many of the histological, genomic, transcriptomic, and molecular features of the parental tumors, including subtype-specific characteristics and intra-tumoral heterogeneity. A major milestone in the field was achieved by Sachs et al., who established a living biobank of long-term expanding BC organoids derived from multiple molecular subtypes, including luminal, HER2-positive, and TNBC [6]. The generated organoids retained the genomic and phenotypic characteristics of the original tumors while enabling functional drug screening and drug sensitivity profiling. This study demonstrated for the first time that BC organoids could be expanded long-term while faithfully preserving disease heterogeneity, thereby providing a robust platform for translational research and precision oncology applications. Since then, numerous studies have confirmed the translational relevance of BC organoids for modeling tumor progression, investigating mechanisms of therapeutic resistance, and predicting treatment responses. The behavior and long-term stability of organoids are strongly influenced by the biochemical and biophysical properties of the ECM, including stiffness, composition, and porosity, which are essential for recreating a physiologically relevant microenvironment that supports cell attachment, hormone responsiveness, proliferation, and morphogenesis [7]. Moreover, culture conditions that promote the maintenance of stem-like cell populations are critical for sustaining organoid expansion and preserving tumor-specific characteristics over time. Despite their considerable potential in precision medicine, PDO models still present several limitations, including variable establishment efficiency, prolonged culture times, elevated costs, and the incomplete representation of stromal, vascular, and immune components of the TME. Furthermore, long-term culture may lead to clonal selection and genetic drift, potentially affecting the fidelity of the original tumor representation. To overcome these challenges, recent efforts have focused on the development of more sophisticated co-culture systems integrating cancer-associated fibroblasts, immune cells, and endothelial components to better reproduce tumor ecosystem dynamics and enhance the physiological relevance of organoid-based models [8]. Overall, BC organoids constitute a powerful translational platform bridging conventional in vitro models and clinical applications. Their ability to preserve the histological, molecular, and functional characteristics of parental tumors makes them particularly attractive tools for drug screening, biomarker discovery, and personalized medicine. Nevertheless, further optimization and standardization of culture conditions are still required to improve reproducibility, scalability, and the accurate representation of TME complexity. While organoid systems provide highly informative ex vivo platforms, they do not fully recapitulate the systemic interactions occurring within a living organism. Consequently, in vivo models remain indispensable for investigating tumor progression, metastatic dissemination, and therapeutic responses in a physiologically integrated context.

### 1.2. Chicken Embryo CAM Model

Alternative platforms are increasingly being explored to bridge the gap between in vitro systems and more complex mammalian models. Among these, the chicken embryo (CE), particularly through the chorioallantoic membrane (CAM) assay, has emerged as a versatile and cost-effective model for investigating tumor growth, angiogenesis, metastatic dissemination, and therapeutic responses under physiologically relevant conditions [9]. The CAM model allows the implantation of human BC cells and patient-derived specimens within a highly vascularized microenvironment, including patient-derived tumor fragments and PDOs, as well as circulating cancer stem cell (cCSC)-derived xenografts, enabling the investigation of tumor–host interactions and neovascularization [10]. Its rapid experimental timeline, low maintenance costs, and reduced ethical constraints make it particularly attractive for preclinical screening and translational oncology research [11]. Beyond its use as a biological model, recent studies have demonstrated the feasibility of integrating advanced imaging technologies within the CE platform. High-frequency ultrasound (HFUS) and magnetic resonance imaging (MRI) have been successfully applied to monitor embryonic development and characterize anatomical structures in ovo, highlighting the potential of this model for non-invasive longitudinal imaging applications [12]. Furthermore, independent studies have demonstrated the feasibility of using advanced imaging modalities within the CAM platform. In particular, MRI has been successfully employed to characterize BC xenografts on the CAM, whereas HFUS has proven valuable for the longitudinal assessment of tumor growth and vascularization in CAM tumor models, further supporting the use of the CE as a versatile multimodal imaging platform [13,14,15]. Therefore, the CE should not be regarded as a replacement for murine models but rather as a complementary intermediate platform capable of facilitating the transition from in vitro studies to mammalian in vivo investigations. When combined with advanced imaging approaches, it may provide a valuable framework for accelerating preclinical evaluation while reducing experimental costs and increasing throughput. Importantly, the use of the CE is consistent with the principles of the 3Rs (Replacement, Reduction, and Refinement), as it can partially replace early-stage animal experiments, reduce the number of rodents required for preliminary screening studies, and minimize animal distress during protocol optimization. Consequently, the integration of CE-based models within preclinical research pipelines may contribute to more ethical and sustainable experimental strategies while maintaining biological complexity and translational relevance [16].

### 1.3. Murine Models

By in vivo models, we refer to experimental systems based on living organisms that allow the investigation of biological processes within a physiologically integrated environment. In BC research, the mouse represents the most widely used and clinically relevant model organism for studying tumor initiation, progression, invasion, metastatic dissemination, and therapeutic responses. Unlike in vitro and ex vivo platforms, murine models enable the evaluation of complex interactions between tumor cells and the host microenvironment, including stromal, vascular, endocrine, and immune components. They represent a critical intermediate step between simplified experimental systems and clinical studies, providing valuable insights into disease biology while facilitating the preclinical assessment of novel therapeutic strategies [17]. Over the past few decades, several mouse-based models have been developed to reproduce different aspects of human BC. These include genetically engineered mouse models (GEMMs), cell line-derived xenografts (CDXs), and patient-derived xenografts (PDXs), each characterized by specific advantages and limitations. Together, these models have substantially contributed to our understanding of BC pathogenesis, metastatic progression, treatment resistance, and therapeutic efficacy.

#### 1.3.1. GEMMs

For investigating early events in breast tumorigenesis, GEMMs are particularly suitable due to their precise genetic control and ability to recapitulate tumor development in a physiologically relevant context. In this model, spontaneous tumor initiation occurs within the appropriate mammary microenvironment from otherwise normal epithelial cells. Tumors arise in situ within the tissue of origin in the presence of an intact immune system, thereby reproducing the sequential stages of tumor initiation, progression, and, in some cases, metastatic dissemination [17]. The first GEMM established to study BC was the MMTV–*Myc* model, in which the oncogene *Myc* was overexpressed under the control of the mouse mammary tumor virus (MMTV) long terminal repeat promoter. This leads to luminal-restricted *Myc* activation in mammary epithelial cells, resulting in increased proliferation and the spontaneous development of luminal-type mammary adenocarcinomas [18]. HER2-enriched mammary carcinomas are commonly modeled using MMTV-driven systems expressing either the rat homolog of *ERBB2 (Neu)* or the human *ERBB2*/HER2 oncogene. These models drive mammary epithelial transformation and faithfully recapitulate key features of HER2-positive BC, including stepwise tumor progression and metastatic potential. In particular, the MMTV–*Neu*/HER2 model has been widely used to study early tumor dissemination and metastatic spread [19,20]. Another highly relevant and widely used GEMM is the MMTV–*PyMT* model, in which the polyoma middle T antigen is expressed under the control of the MMTV promoter, leading to rapid and highly penetrant mammary tumor formation. This model exhibits multistage tumor progression that closely resembles human BC, including hyperplasia, adenoma/mammary intraepithelial neoplasia, and carcinoma, followed by efficient metastasis, particularly to the lungs. Owing to its aggressive phenotype and well-defined progression kinetics, the MMTV–*PyMT* model has been extensively used to investigate tumor progression, epithelial plasticity, and metastatic mechanisms in BC [21,22]. The primary strength of GEMMs lies in their ability to facilitate mechanistic studies through precise genetic manipulation, allowing tumors to arise spontaneously within their tissue of origin in the presence of an intact immune system. Consequently, this model preserves critical interactions between tumor cells and the surrounding microenvironment, providing valuable insights into tumor initiation, progression, and metastatic dissemination. Furthermore, GEMMs enable the investigation of specific oncogenic pathways and their contribution to BC development in a physiologically relevant setting. Despite these advantages, the generation and maintenance of GEMMs are labor-intensive, time-consuming, and costly. In addition, these models do not always fully recapitulate the genetic and phenotypic heterogeneity observed in human BC. A particular limitation concerns the modeling of hormone receptor-positive (HR+) disease, as many commonly used GEMMs, including MMTV-driven models, tend to lose hormone receptor expression during tumor progression and frequently evolve toward more aggressive HER2-positive or triple-negative phenotypes. Although a limited number of models, such as Stat1-deficient mice, can develop estrogen receptor-positive tumors, these lesions often display slow growth rates and limited metastatic potential, thereby restricting their utility for studying advanced and metastatic HR+ BC [21].

#### 1.3.2. CDXs

To overcome some of the limitations associated with GEMMs, xenograft-based approaches have been widely adopted in BC research. Among these, CDXs represent one of the most established and extensively used in vivo models. CDXs are generated through the implantation of established human BC cell lines into immunocompromised mice, either orthotopically into the mammary fat pad or ectopically at subcutaneous sites [21]. The use of human cancer cells enables the investigation of tumor growth, metastatic behavior, and therapeutic responses in a living organism while maintaining experimental reproducibility and relatively low costs. The first BC xenograft model was reported by Ozello and colleagues in 1974, who successfully implanted the TNBC cell line BT-20 into athymic nude mice. Although metastatic dissemination was not observed, this study demonstrated the feasibility of propagating human breast tumors in immunodeficient hosts and laid the foundation for the development of modern xenograft models [23]. Subsequent studies expanded the range of transplantable BC cell lines and demonstrated that tumor growth, metastatic potential, and therapeutic responses could vary substantially according to the molecular characteristics of the implanted cells. One of the main advantages of CDX models is their ease of establishment and scalability, which makes them particularly suitable for preclinical drug testing and mechanistic studies. Furthermore, the availability of well-characterized BC cell lines representing distinct molecular subtypes, including luminal, HER2-positive, and TNBCs, has facilitated the investigation of subtype-specific biological processes, therapeutic weaknesses, and treatment responses. Recent multi-omics analyses have further refined the molecular characterization of BC cell lines, improving their utility as representative models of specific disease subtypes and supporting their application in translational and preclinical research [24,25]. Owing to its reproducibility and ease of manipulation, this model has been extensively employed in preclinical studies to investigate tumor biology, metastatic behavior, therapeutic responses, and mechanisms of drug resistance across different BC subtypes. Furthermore, advances in molecular profiling, genome editing, and in vivo imaging technologies have expanded the utility of CDX models, enabling more sophisticated functional studies and accelerating the preclinical evaluation of novel therapeutic strategies [26]. However, CDXs also present important limitations. Prolonged adaptation of cancer cell lines to in vitro culture conditions often results in the loss of tumor heterogeneity and the selection of highly proliferative cellular clones. Consequently, CDX tumors frequently fail to fully recapitulate the genomic complexity, cellular diversity, and microenvironmental interactions observed in patient tumors. Moreover, because xenografts are typically established in immunodeficient mice, the contribution of the immune system to tumor progression and therapeutic response cannot be adequately evaluated. These limitations have driven the development of PDX models, which more faithfully preserve the histological and molecular characteristics of human breast tumors.

#### 1.3.3. PDXs

To overcome the limited heterogeneity and clinical representativeness of CDXs, PDX models have emerged as valuable platforms for translational BC research. PDXs are generated through the direct implantation of freshly resected patient tumor specimens into immunocompromised mice. By avoiding prolonged in vitro culture, this model preserves many of the histological, molecular, genomic, and transcriptomic characteristics of the original tumor, including intra-tumoral heterogeneity and subtype-specific features. The translational potential of PDXs in BC research was first demonstrated by DeRose et al., who established a collection of patient-derived tumor grafts that faithfully reproduced the pathological, molecular, and metastatic characteristics of the corresponding patient tumors across several passages [27]. Subsequent studies further validated the ability of PDXs to capture the biological diversity of BC and highlighted their utility for biomarker discovery, therapeutic testing, and the study of treatment resistance [28,29]. Over the last decade, PDX models have become increasingly important in precision oncology. The establishment of large-scale international PDX biobanks and repositories, encompassing HR+, HER2-positive, and TNBCs, has enabled comprehensive investigations of tumor evolution, therapeutic vulnerabilities, and clinically relevant drug response patterns. In parallel, the integration of multi-omics and molecular profiling approaches has demonstrated that PDXs can stably preserve patient-specific mutational landscapes, copy number alterations, gene expression programs, and epigenetic features across serial passages. These advances have further strengthened the value of PDXs as clinically relevant models for preclinical drug development, co-clinical trial design, biomarker discovery, and the identification of mechanisms underlying therapeutic resistance [26,30]. The availability of well-characterized PDX collections has also facilitated the investigation of intratumoral heterogeneity, clonal evolution, and subtype-specific biological vulnerabilities. More recently, the development of next-generation platforms combining PDO and matched PDX models has enabled complementary ex vivo and in vivo studies within the same patient-derived biological system. In parallel, the emergence of humanized PDX models, generated through the reconstitution of components of the human immune system in recipient mice, has provided new opportunities to investigate tumor–immune interactions and evaluate immunotherapeutic strategies.

**Figure 1:**
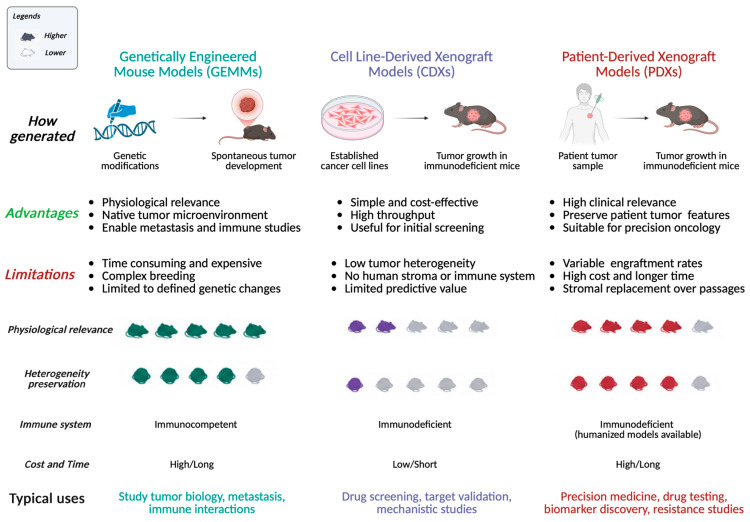
Murine models in BC research. Overview of the main murine models used in BC research, with their key features, applications, and limitations (created with BioRender.com).

Despite these advances, several challenges remain. Engraftment efficiency varies considerably among BC subtypes and is generally higher for aggressive tumors such as TNBC, potentially introducing selection bias within PDX repositories. Furthermore, progressive replacement of human stromal components by murine counterparts during serial passaging may alter microenvironmental interactions. Finally, the development of robust models faithfully reproducing HR+ disease, metastatic dormancy, and late disease recurrence remains an important unmet need in BC research. Nevertheless, ongoing efforts to improve PDX platforms, particularly through humanized systems and integrated patient-derived model platforms, continue to enhance their value in translational and precision oncology [21]. Figure 1 summarizes the main murine models used in BC research and highlights their key characteristics and limitations.

### 1.4. Comparative Strengths and Limitations of Preclinical Breast Cancer Models and Emerging Hybrid Platforms

Each preclinical BC model has distinct advantages and limitations, making it suitable for addressing specific biological and translational questions. Consequently, the choice of the most appropriate experimental platform should be based on the study’s ultimate objective in terms of the biological process under investigation, the level of biological complexity required, and the imaging modalities to be used. Table 1 summarizes the principal characteristics, optimal applications, strengths, limitations, and imaging opportunities of the major preclinical BC models discussed in this review.

Collectively, these experimental platforms should be regarded as complementary rather than mutually exclusive. Three-dimensional organoids and PDOs provide highly controlled human-derived systems for investigating tumor biology, intratumoral heterogeneity, and therapeutic responses, while enabling high-throughput drug screening. The CAM model represents an intermediate platform between advanced in vitro systems and mammalian models, allowing rapid evaluation of tumor growth, angiogenesis, invasion, and treatment response within a vascularized in vivo environment while supporting the principles of the 3Rs. GEMMs, CDXs, and PDXs progressively increase biological complexity by incorporating tumor–host interactions, metastatic dissemination, immune components, and pharmacological responses within a whole-organism context. However, no single model can fully recapitulate the complexity of human BC. Therefore, current research is increasingly moving toward integrated and sequential experimental workflows, in which complementary models are combined according to the specific stage of preclinical investigation, from mechanistic studies and drug screening to biomarker discovery and translational validation. Beyond conventional organoid cultures, emerging hybrid platforms integrating PDOs, microfluidic technologies, and in vivo imaging approaches represent promising strategies to bridge controlled human-derived models with physiologically relevant systems. Microfluidic organ-on-chip devices enable precise manipulation of the TME, allowing the investigation of dynamic cell–cell interactions, immune cell infiltration, angiogenesis, and drug delivery under controlled conditions. When combined with PDOs, these systems preserve patient-specific tumor characteristics while providing reproducible platforms for functional studies and therapeutic screening. Furthermore, the integration of organoid-based models with longitudinal in vivo imaging in orthotopic or PDX models enables the assessment of tumor progression, metastatic dissemination, and treatment response across multiple biological scales [31]. Such multimodal experimental frameworks, combined with advanced imaging technologies, represent a promising approach to improve the predictive value and translational relevance of preclinical BC research.

## 2. Imaging Modalities in Preclinical Breast Cancer

In preclinical BC research, advanced imaging modalities provide complementary insights across multiple biological scales, enabling the investigation of tumor progression from whole-body tumor monitoring to single-cell interactions within the TME [32]. No single imaging technique is capable of fully capturing the spatial, temporal, and molecular complexity of BC progression; therefore, multimodal approaches are increasingly employed to integrate anatomical, functional, and cellular information [33]. Among the most widely used imaging platforms are optical imaging (OI), HFUS, MRI, and positron emission tomography/computed tomography (PET/CT), each providing distinct yet complementary information across experimental platforms. These modalities enable, respectively, longitudinal monitoring of tumor growth, real-time assessment of vascular and mechanical properties, high-resolution anatomical and functional characterization, and quantitative evaluation of tumor metabolism and metastasis dissemination. At a higher cellular resolution, intravital microscopy (IVM) provides unique insights into dynamic tumor–stroma and tumor–immune interactions within the living microenvironment [34]. Unlike conventional imaging-centric reviews, this section emphasizes the multiscale integration of imaging readouts with tumor biology across in vitro and murine BC models.

### 2.1. OI

OI approaches are widely employed in preclinical BC research due to their high sensitivity, relatively low cost, and ability to provide dynamic visualization of tumor-associated biological processes in vivo. Among the most used OI modalities are bioluminescence imaging (BLI) and fluorescence imaging (FLI), both extensively applied for longitudinal monitoring of tumor progression, metastatic dissemination, and therapy response in murine BC models.

BLI represents the most adopted modality for in vivo tumor monitoring [35]. This technique relies on luciferase-expressing tumor cells to generate light emission following substrate administration [36]. Owing to its non-invasive nature and relatively low cost, BLI has become a broadly adopted approach for longitudinal monitoring of tumor burden in murine BC models, enabling real-time assessment of primary tumor growth, metastatic dissemination, and therapeutic response over time [37]. Recent advances in BLI have supported the detection of minimal residual disease following chemotherapy in murine BC models, highlighting its sensitivity for therapy response monitoring [38]. These approaches allow temporal tracking of tumor cell survival after chemotherapeutic interventions. The ability of BLI to detect residual tumor burden at very low cell numbers underscores its value in preclinical evaluation of therapeutic efficacy. In addition, BLI has been extensively used to monitor metastatic dissemination in aggressive BC subtypes, particularly in models characterized by high metastatic potential [39]. Beyond conventional tumor monitoring, recent developments in reporter-based OI have expanded the applications of BLI toward the investigation of more complex biological processes. Bioluminescent approaches have been employed to track circulating tumor cells and metastatic dissemination in vivo [40]. Moreover, NanoLuciferase-based reporter platforms have recently provided real-time visualization of extracellular vesicle kinetics in murine BC models, further extending the role of BLI in studying tumor progression and intercellular communication within the TME [41]. Despite its high sensitivity and ease of use, BLI remains limited by low spatial resolution and reduced tissue penetration, which may hinder accurate anatomical localization of deep lesions.

In addition to BLI, FLI has become an important OI approach in preclinical BC research, particularly for the visualization of cellular and molecular processes with higher spatial resolution [42]. Fluorescent probes and near-infrared (NIR) fluorophores have been extensively employed for tumor detection, image-guided surgery, lymphatic mapping, and investigation of TME interactions [43,44]. Compared with BLI, FLI provides higher spatial resolution and multiplexing capability, enabling more detailed spatial mapping of cellular and molecular events, although tissue autofluorescence and limited penetration depth may compromise signal specificity in deeper tissues [45]. Recent developments in NIR-II FLI have further improved tissue penetration and signal-to-background ratio, expanding the applicability of OI toward deep-tissue visualization with improved signal-to-background performance in preclinical cancer models [46].

Overall, OI modalities provide highly sensitive and versatile tools for real-time investigation of tumor biology in preclinical BC models, particularly when integrated with complementary anatomical imaging techniques.

### 2.2. HFUS

HFUS is a non-invasive imaging modality employed in preclinical BC research for real-time evaluation of tumor growth and vascularization within the broader framework of preclinical molecular imaging approaches aimed at bridging experimental models and translational oncology applications [47]. Compared with conventional clinical ultrasound systems, HFUS provides higher spatial resolution and is extensively used for rapid, sensitive, and cost-effective monitoring of tumor grafts in small-animal models [48]. Advanced ultrasound approaches, including high-frequency Doppler and contrast-enhanced ultrasound (CEUS), have further expanded the role of HFUS in the assessment of tumor-associated microcirculation and vascular heterogeneity in metastatic BC models. These techniques enable high-resolution visualization of perfusion dynamics in metastatic lymph nodes, providing valuable functional information on tumor vascular remodeling [49]. More recently, HFUS-based elastography techniques have allowed the quantitative assessment of tumor stiffness and tissue heterogeneity in vivo, providing biomechanical biomarkers potentially associated with tumor progression and therapeutic response in preclinical cancer models [50]. Despite its accessibility and real-time imaging capability, HFUS remains limited by operator dependency and reduced penetration depth in deeper tissues, but it complements other imaging modalities by providing high-resolution anatomical and functional information in real time, particularly in longitudinal studies of tumor progression.

### 2.3. MRI

MRI is a high-resolution, non-invasive imaging modality mostly used in preclinical BC research for detailed anatomical and functional characterization of tumor growth and the TME [51]. In murine BC models, MRI enables precise longitudinal assessment of tumor volume, vascular architecture, and response to therapy, particularly in orthotopic and metastatic settings [52]. Beyond volumetric assessment, MRI provides critical insights into tumor heterogeneity, necrosis, and tissue microenvironmental changes. Multiparametric MRI integrates complementary anatomical and functional sequences, enabling a comprehensive characterization of tumor biology. Advanced MRI techniques, including diffusion-weighted imaging (DWI) and dynamic contrast-enhanced (DCE) MRI, provide quantitative biomarkers of tumor cellularity, vascular permeability, and treatment-induced changes in the TME [53,54]. Importantly, MRI represents a key translational imaging modality, as many imaging biomarkers used in preclinical BC models are directly applicable in clinical oncology [51], complementing optical and ultrasound imaging modalities through high-resolution anatomical and functional information with established translational relevance.

### 2.4. PET/CT

PET/CT is a highly sensitive, non-invasive imaging modality used for the quantitative assessment of tumor proliferation, metabolism, disease growth or dissemination, and treatment response [55,56]. PET/CT enables the detection of metabolic alterations preceding anatomical changes, providing early functional insights into tumor activity and progression [57]. In murine BC models, fluorodeoxyglucose (FDG)–PET is commonly employed to evaluate glucose metabolism, allowing precise longitudinal monitoring of primary tumor growth and metastatic spread across the whole body [58]. This metabolic sensitivity makes PET/CT particularly valuable in assessing early therapeutic response and minimal residual disease in preclinical studies [59]. Beyond metabolic imaging, PET/CT has been increasingly applied to investigate tumor heterogeneity and microenvironmental changes. This includes radiotracers targeting proliferation, hypoxia, and receptor expression [60]. These advanced applications support a more comprehensive molecular characterization of BC beyond glucose metabolism alone. Importantly, PET/CT represents a key translational imaging modality, as metabolic imaging biomarkers are generally used in clinical oncology for staging, prognosis, and therapy monitoring [61]. The integration of PET-derived functional information with CT-based anatomical localization provides a powerful multimodal approach for bridging preclinical findings with clinical BC management [62]. Despite its high sensitivity and quantitative capabilities, PET/CT is limited by relatively low spatial resolution compared with MRI and high operational costs, which may restrict its routine use in small-animal longitudinal studies [63].

### 2.5. Multimodal Integration of Imaging Approaches

The imaging modalities discussed above provide complementary information across different spatial, temporal, and biological scales, highlighting the importance of multimodal approaches in this type of preclinical research. While BLI and HFUS are particularly suited for rapid longitudinal monitoring and real-time functional assessment, MRI and PET/CT provide deeper anatomical, metabolic, and quantitative characterization of tumor progression and therapeutic response. Therefore, the integration of multiple imaging platforms represents an increasingly valuable strategy for bridging molecular, cellular, and whole-body information within translational BC models [33]. As summarized in Table 2, these imaging modalities are compared in terms of their main characteristics, advantages, limitations, and translational potential, further emphasizing their complementary roles in multiscale preclinical BC research.

**Figure 2:**
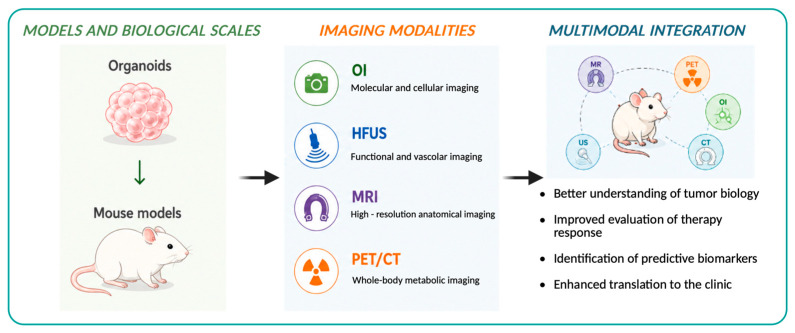
Multimodal imaging approaches in preclinical BC research. Schematic comparison of the complementary roles of OI, HFUS, MRI, and PET/CT in the multiscale characterization of BC models. The integration of these imaging modalities enables the combined assessment of molecular, functional, anatomical, and metabolic features, improving translational imaging workflows and biomarker development (created with BioRender.com).

Despite these complementary strengths, each imaging modality presents inherent limitations. Consequently, multimodal and hybrid imaging strategies are increasingly being adopted to overcome the shortcomings of individual techniques. The combination of OI with CT, for example, as well as hybrid PET/CT and PET/MRI platforms, allows for the integration of highly sensitive molecular and functional information with detailed anatomical characterization [64,65]. For example, OI approaches provide highly sensitive detection of tumor-associated molecular signals, whereas CT and MRI enable precise anatomical localization and structural characterization of the same lesions. Similarly, hybrid PET/MRI systems combine metabolic information with high-resolution soft-tissue imaging, improving the assessment of tumor heterogeneity and therapy-induced biological changes and the correlation between imaging phenotypes and tumor molecular features. In this context, PET/MRI-derived functional parameters have also been shown to correlate with immunohistochemical markers and to contribute to the prediction of BC molecular subtypes, supporting the translational value of hybrid imaging approaches [66]. In particular, hybrid imaging systems represent a powerful translational bridge between preclinical research and clinical oncology, facilitating the development of imaging biomarkers with direct clinical applicability. Figure 2 provides a schematic overview of multimodal imaging strategies in preclinical BC models, highlighting how complementary imaging modalities can be integrated to achieve a comprehensive multiscale characterization of tumor biology.

## 3. IVM: Bridging Cellular Dynamics and Whole-Body Imaging

IVM has emerged as a powerful imaging approach for investigating dynamic biological processes within living tissues at single-cell resolution. In contrast to non-invasive whole-body imaging modalities, including OI, HFUS, MRI, and PET, which primarily provide anatomical, functional, or molecular information at the organ or whole-body level, IVM enables the direct visualization of cellular behavior within the TME. This unique capability allows real-time monitoring of interactions among tumor cells, stromal populations, immune cells, ECM components, and the vasculature during disease progression. The introduction of advanced fluorescence microscopy, together with mammary imaging windows and dorsal skinfold chambers, has enabled longitudinal imaging of the same tumor over days or weeks, providing continuous observation of tumor progression, treatment response, and metastatic dissemination within intact physiological environments [67,68]. Combined with fluorescent reporters, genetically engineered mouse models, and cell-specific labeling strategies, IVM allows quantitative assessment of tumor cell migration, proliferation, apoptosis, vascular permeability, ECM remodeling, and immune cell recruitment at cellular resolution, providing information that cannot be obtained by conventional macroscopic imaging alone [69]. One of the major contributions of IVM has been the characterization of metastatic progression. Direct visualization of tumor cell dissemination has demonstrated that metastasis is organized through dynamic interactions between cancer cells and the surrounding microenvironment, highlighting the essential roles of tumor-associated macrophages, cancer-associated fibroblasts, endothelial cells, and ECM remodeling in local invasion and intravasation [70,71]. IVM has also significantly advanced the study of tumor angiogenesis and cancer immunology. Multiphoton microscopy enables real-time visualization of vascular architecture, blood flow, vascular permeability, and perivascular interactions, improving our understanding of tumor perfusion and supporting the development of vascular-targeted therapies [72]. In parallel, fluorescent immune reporters have enabled longitudinal tracking of immune cell recruitment, migration, and activation, providing mechanistic insights into immune surveillance, immune evasion, and responses to immunotherapy [73]. Beyond its applications in tumor biology, IVM represents a valuable platform for evaluating therapeutic efficacy by enabling real-time visualization of drug delivery, vascular responses, cellular apoptosis, and the emergence of therapy-resistant tumor cell populations. These mechanistic observations complement imaging biomarkers derived from MRI, PET, and OI, facilitating the identification of predictive biomarkers and novel therapeutic targets. Despite its exceptional spatial resolution, IVM has important limitations, including limited imaging depth, restricted field of view, the need for surgical implantation of imaging windows, and technically demanding experimental procedures. Consequently, it should be regarded as complementary rather than an alternative to non-invasive imaging modalities. Within the multiscale imaging framework of BC, IVM holds a unique intermediate position between high-resolution ex vivo microscopy and whole-body imaging. While organoids and other 3D culture systems provide controlled experimental models and MRI or PET enable longitudinal assessment of tumor burden and therapeutic response in vivo, IVM bridges these approaches by capturing dynamic cellular processes in their native microenvironment. Integrating IVM with non-invasive imaging therefore provides a comprehensive multiscale characterization of BC, combining cellular, molecular, functional, and anatomical information to improve the translational relevance of preclinical models.

## 4. Translational Applications in Precision Oncology

### 4.1. In Vivo Imaging-Guided Drug Response, Metastasis Tracking, and Therapeutic Resistance

The integration of advanced imaging approaches with 3D and in vivo models enables quantitative and longitudinal monitoring of the therapeutic response, providing dynamic information on tumor progression, treatment efficacy, and the emergence of resistant phenotypes. By enabling the noninvasive assessment of biological processes such as proliferation, apoptosis, metabolic activity, and drug distribution, imaging-guided strategies complement conventional endpoint analyses and improve the prediction of therapeutic outcomes in preclinical models. The validation of imaging biomarkers in corresponding in vivo models remains essential to confirm that the results obtained in advanced 3D systems accurately reflect the complexity of intact tissue architecture and systemic physiology, thereby strengthening their translational relevance. Several studies have demonstrated the potential of imaging-based approaches for monitoring treatment response and resistance mechanisms in BC models.

Usui et al. employed EGFR-positive BC CDX models to evaluate a ^64^Cu-labeled cetuximab immuno-PET tracer for the detection of primary tumors and sentinel lymph node metastases. The study demonstrated high tumor-specific uptake and accurate visualization of metastatic lymph nodes, highlighting the potential of molecular imaging approaches to improve early detection of metastatic dissemination and to support image-guided therapeutic strategies in BC [74]. Li et al. developed a CD47-targeted dual-modality PET/NIR-II fluorescence imaging probe and evaluated its performance in orthotopic BC CDX models. The combined imaging strategy enabled sensitive tumor detection, precise anatomical localization, and intraoperative visualization of metastatic lesions, demonstrating how hybrid imaging approaches can enhance both tumor characterization and image-guided surgical interventions in preclinical BC research [75]. In the preclinical study conducted by Tao et al., the radiotracer ^68^GaGA-NODAGA-ADAPT6 was evaluated in mouse models with xenografts of HER2-positive HCC1954 and HER2-negative MDA-MB-468 tumors. Biodistribution and micro-PET/CT imaging studies demonstrated high in vivo stability, rapid clearance from the bloodstream, and selective accumulation in HER2-expressing tumors. Uptake was significantly higher in HCC1954 xenografts compared to HER2-negative controls, while pretreatment with trastuzumab markedly reduced tracer accumulation, confirming specific receptor binding. The ability of ^68^GaGA-NODAGA-ADAPT6 to sensitively detect changes in HER2 accessibility following receptor blockade highlights its potential as a non-invasive biomarker for assessing target engagement, monitoring therapeutic response, and identifying dynamic changes in HER2 expression during anti-HER2 therapy. These results support the use of ADAPT6-based PET imaging as a valuable tool for the longitudinal assessment of treatment efficacy and patient stratification in HER2-positive BC [76]. Taken together, these studies demonstrate that molecular imaging is evolving beyond simple tumor detection, moving toward the quantitative assessment of target expression and pharmacodynamic changes during therapy. Despite differences among imaging probes in terms of molecular targets, their use consistently demonstrates that it is possible to detect early functional changes before measurable anatomical responses occur, highlighting the potential of imaging biomarkers for treatment stratification and adaptive therapeutic monitoring.

Bartsch et al. further demonstrated the value of correlative multimodal imaging in BC xenograft models through the integration of multiparametric PET/MRI, histopathology, and matrix-assisted laser desorption/ionization imaging mass spectrometry (MALDI-IMS). Their workflow combined simultaneous PET and functional MRI acquisitions, including diffusion-weighted imaging, intravoxel incoherent motion MRI, DCE-MRI, blood oxygen level-dependent MRI, and glucose-sensitive CEST imaging, with ex vivo molecular and proteomic analyses. Using BC xenografts as a proof-of-concept model, the authors demonstrated the feasibility of correlating in vivo imaging biomarkers of metabolism, perfusion, cellularity, and hypoxia with ex vivo molecular information across different spatial scales. The study showed that multimodal imaging can effectively capture intratumoral heterogeneity and generate biologically meaningful imaging signatures, providing a framework for the development of radioproteomic biomarkers with potential applications in therapy stratification, treatment monitoring, and translational precision oncology [77]. Song et al. investigated the biological effects of the selective HER2 tyrosine kinase inhibitor, tucatinib, using complementary molecular imaging approaches in both CDX and PDX models of HER2-positive BC. Specifically, HER2-positive BT474 xenografts and BCM-3472 PDX tumors were established in immunodeficient mice and treated with tucatinib. Longitudinal PET imaging was performed using ^18^F-fluorothymidine (FLT)-PET to assess tumor proliferation, ^18^F-fluoromisonidazole (FMISO)-PET to quantify hypoxia, and ^89^Zr-pertuzumab-PET to evaluate HER2 expression and receptor availability before treatment initiation and at multiple time points during therapy. Tucatinib treatment induced significant reductions in both proliferative activity and tumor hypoxia in CDX and PDX models. In BT474 xenografts, HER2-targeted PET imaging additionally revealed a significant reduction in HER2 expression following treatment, whereas no significant HER2 modulation was observed in the BCM-3472 PDX model, highlighting biologically relevant differences between experimental systems. Overall, the study demonstrated that multiparametric PET imaging can non-invasively capture early tucatinib-induced changes in cells and the TME before substantial anatomical alterations occur, supporting the use of molecular imaging biomarkers for longitudinal treatment monitoring and response prediction in HER2-positive BC [78]. Mansur et al. investigated the therapeutic efficacy of the combination of trastuzumab and the PARP inhibitor, niraparib, in HER2-positive BC using both a CDX model (BT474) and a PDX model (BCM-3472). To non-invasively monitor early biological changes induced by treatment, the authors used ^18^F-FLT-PET/CT at baseline and during drug administration. Both in vitro and in vivo, the combined therapy produced greater tumor growth inhibition than monotherapy, with tumor regression observed only in the groups receiving the combination treatment. The study demonstrated that ^18^F-FLT-PET can serve as a sensitive imaging biomarker for the early assessment of response to HER2-targeted combination therapies in clinically relevant HER2-positive PDX models, highlighting its potential utility for treatment monitoring and therapeutic optimization [79]. Taken together, these multimodal imaging studies demonstrate that the combination of complementary imaging biomarkers allows for a more comprehensive characterization of the therapeutic response than any single modality used alone. While PET-derived biomarkers sensitively detect early metabolic and proliferative changes, MRI provides spatial and functional information on tumor perfusion and heterogeneity. Their integration therefore represents a promising strategy for improving response prediction in preclinical precision oncology, although standardized acquisition and analysis protocols remain necessary for broader translational implementation.

In the DCIS-MIND (mouse-intraductal) biobank study performed by Hutten et al., tumor progression was primarily monitored using longitudinal BLI, which allowed for the precise detection of intraductal growth and invasive spread. FLI of whole specimens and confocal reconstruction, used in a complementary manner, provided spatial resolution of ductal architecture and invasion patterns, facilitating the identification of the biological determinants of DCIS progression [80].

The authors generated a patient-derived DCIS-MIND model that represents a specialized form of PDX in which human DCIS cells are injected directly into the mammary ducts of immunodeficient mice. Although distinct from classical subcutaneous or orthotopic PDXs, this intraductal approach preserves the ductal microenvironment and enables physiologically relevant modeling of in situ growth and invasive progression. Within the DCIS-MIND intraductal PDX framework, longitudinal BLI and high-resolution FLI of whole specimens provide sensitive insights into intraductal growth, ductal architecture, and early invasive escape, enabling direct alignment of imaging endpoints between patient-derived lesions and murine hosts. This model integrates perfusion- and metabolism-based parameters acquired using advanced in vivo modalities such as DCE-MRI, DWI, and PET employed in invasive BC models. It is important to note that this model bridges a fundamental gap between conventional xenograft systems and clinical DCIS, enabling a longitudinal study of the transition from pre-invasive to invasive disease. However, before its translational potential can be fully established, broader validation across all molecular subtypes of BC will be necessary.

Griessinger et al. used a BC GEMM model that replicates the multistage progression from precancerous lesions to invasive disease and combined multi-tracer PET with high-resolution MRI to study intratumoral heterogeneity and tumor evolution in vivo. The imaging protocol included ^18^F-FDG-PET to assess glucose metabolism, ^18^F-FLT-PET to assess cell proliferation, and RGD-based PET targeting integrin αvβ3 expression as a marker of neoangiogenesis. These molecular imaging data were integrated with anatomical and functional MRI and analyzed to identify distinct intratumoral regions characterized by specific biological features. The study demonstrated that different stages of BC progression exhibited unique imaging characteristics, allowing for the distinction between premalignant lesions, early-stage invasive tumors, and more advanced-stage disease. Furthermore, multiparametric imaging revealed marked spatial heterogeneity within individual tumors, identifying coexisting regions with distinct metabolic, proliferative, and angiogenic phenotypes that would not have been detectable using a single imaging modality. These results highlight the value of advanced multimodal imaging in genetically engineered BC models for the longitudinal monitoring of tumor progression, the study of intratumoral heterogeneity, and the development of imaging biomarkers with potential translational applications in precision oncology [81].

Madonna et al. used a GEMM model of HER2/*Neu*-positive BC to study the metabolic adaptations associated with residual disease and tumor recurrence following HER2-targeted therapy. Unlike conventional xenograft models, this GEMM model reproduces the full spectrum of disease progression, including tumor regression, residual disease, and spontaneous recurrence within an intact TME. Using longitudinal in vivo metabolic imaging combined with molecular analyses, the authors monitored metabolic changes throughout treatment and disease progression. Importantly, imaging enabled the noninvasive detection of tumor metabolic alterations during the transition from residual disease to recurrence, identifying lipid metabolism as a potential therapeutic vulnerability associated with treatment resistance. Overall, these results further underscore the value of GEMMs as translational platforms for molecular imaging studies, thereby facilitating the discovery of imaging biomarkers and therapeutic vulnerabilities associated with treatment response, residual disease, and recurrence [82]. These studies highlight the unique strengths of GEMMs for longitudinal imaging of tumor evolution, as they preserve the tumor’s endogenous development and immune interactions. However, their relatively long turnaround times, higher costs, and limited representation of patient-specific genomic diversity continue to limit their large-scale application, underscoring the complementary value of xenograft-based platforms.

Multiple studies have integrated PDXs with in vivo imaging modalities to validate drug responses observed in PDOs. Classical PDX models are routinely monitored through MRI, PET, and BLI to quantify tumor burden, perfusion, metabolism, and therapeutic response, as highlighted in recent translational frameworks. Mini-PDX systems further enable rapid in vivo validation of PDO-derived predictions, while integrated PDX/patient-derived xenograft organoid (PDxO) platforms extend this approach to iterative drug discovery pipelines. Collectively, these models provide the in vivo imaging endpoints, volumetric MRI, metabolic PET, and longitudinal BLI that anchor PDO-based findings within whole-organism physiology.

Guillen et al. developed a large biobank of PDX and organoids of BC, integrating imaging modalities such as MRI and PET to validate pharmacological responses. They demonstrated that imaging biomarkers identified in organoids, such as metabolic shifts and proliferation dynamics, correlated with therapeutic efficacy in murine models, supporting the predictive value of imaging-guided drug screening [83].

Cui et al. further demonstrated the translational value of combining PDO with mini-PDX models. They established PDOs from BC patient biopsies, which preserved tumor heterogeneity, receptor status, and microenvironmental features. These PDOs were used for rapid in vitro drug testing with high-content imaging to evaluate proliferation, apoptosis, and morphological changes. In parallel, mini-PDX models were generated by implanting small tumor fragments into immunodeficient mice, allowing for short-term in vivo validation of drug responses. Imaging modalities such as MRI, PET, and BLI were employed to monitor tumor growth, vascularization, and metabolic activity in the murine setting. The combined platform demonstrated that drug responses observed in PDOs correlated strongly with outcomes in mini-PDX models, confirming translational relevance. PDOs provided rapid screening within days, while mini-PDX offered systemic validation within weeks. The observed concordance between ex vivo and in vivo responses supports the integration of multimodal imaging for patient-specific therapeutic testing [84]. Guillen et al. established a comprehensive platform integrating PDXs and matched PDOs from advanced, treatment-resistant, and metastatic BC, including TNBC, endocrine-resistant, and HER2-positive tumors. The authors generated a large biobank of paired in vivo and in vitro models and demonstrated that PDxOs retained the histological, genomic, transcriptomic, and pharmacological characteristics of their parental tumors and corresponding PDXs. High-throughput drug screening was performed using PDxOs cultured in 3D conditions, followed by in vivo validation in the matched PDX models. Drug responses observed in organoids were highly concordant with those obtained in vivo, supporting the predictive value of the platform for therapeutic testing. Importantly, the authors demonstrated the translational potential of this approach through a precision oncology case study in a patient with metastatic TNBC. Functional drug screening identified eribulin as a highly effective therapy, and subsequent clinical treatment resulted in a complete response and substantially prolonged progression-free survival. By combining the scalability of organoid-based screening with the biological fidelity of PDX models, this integrated PDxO–PDX platform provides a powerful framework for drug discovery, biomarker development, and functional precision medicine in BC [83]. Beyond its utility for drug screening, this study established the conceptual basis for a multiscale translational workflow in which PDO, advanced 3D culture systems, and matched xenograft models can be combined with functional imaging approaches to investigate therapeutic response, resistance mechanisms, and tumor evolution across different levels of biological complexity. In summary, these integrated PDO-PDX workflows highlight that neither platform, on its own, is enough for precision oncology applications. PDO models offer scalability and rapid functional screening, while PDX models preserve systemic physiology and enable longitudinal imaging of the therapeutic response. Their combined use therefore represents a complementary strategy for translating patient-specific drug sensitivity into clinically relevant imaging biomarkers.

### 4.2. 3D Experimental Platforms

Recent advances in imaging technologies have expanded the role of advanced 3D models of BC, enabling high-resolution visualization of tumor architecture, interactions between cells and the microenvironment, and treatment-induced changes. In this context, vascularized BC spheroids have emerged as a promising intermediate platform that bridges in vitro and in vivo models, providing a physiologically relevant system for the imaging-guided assessment of therapeutic response and resistance mechanisms.

Ascheid et al. have introduced a platform based on vascularized BC spheroids, analyzed using high-resolution light-sheet microscopy, which enables a 3D quantitative assessment of vascular architecture, drug penetration, and therapeutic effects targeted at the microenvironment. This imaging-based system serves as a mesoscopic bridge between conventional PDO assays and in vivo functional imaging, allowing for the capture of microenvironmental dynamics that would otherwise be inaccessible in standard organoid cultures. Within a multiscale PDO → PDX → imaging framework, the vascularized spheroid platform developed by Ascheid et al. adds an intermediate level rich in imaging information, leveraging fluorescence light-sheet microscopy to quantify drug penetration, vascular remodeling, and microenvironment-dependent responses in 3D. This mesoscopic imaging approach integrates high-content in vitro phenotyping of PDOs with downstream in vivo modalities, such as DCE-MRI, DWI, and PET, strengthening the translational continuum from controlled microenvironmental perturbations to whole-organism therapeutic response [85]. Compared to conventional organoids, vascularized spheroid models faithfully reproduce drug transport, endothelial interactions, and spatial gradients within the TME. However, they remain simplified systems that are unable to replicate the systemic immune and endocrine influences present in vivo, which justifies their use as intermediate models rather than substitutes.

In vitro organoid systems exposed to prolonged drug treatment can reproduce adaptive changes associated with resistance, including ECM remodeling, altered tissue architecture, reduced drug penetration, and metabolic reprogramming. Advanced OI approaches, including fluorescence lifetime imaging microscopy (FLIM), enable the detection of these dynamic alterations at high spatial resolution, facilitating the identification of early resistance-associated phenotypes. Complementary in vivo imaging modalities such as MRI, PET, CEUS, and elastography further capture resistance-related changes in vascularization, metabolism, and tissue mechanical properties that arise within the TME. By integrating imaging biomarkers across organoid and murine models, researchers can better elucidate the biological mechanisms underlying therapeutic escape and identify novel strategies to overcome treatment resistance. Consistent with this concept, Lamouline et al. employed co-culture organoid systems and murine imaging approaches to investigate BC bone metastasis and demonstrated that stromal remodeling and hypoxia within the metastatic microenvironment can promote therapy resistance, highlighting the importance of imaging-guided evaluation of TME interactions [86]. The living biobank established by Sachs et al. provides a powerful experimental framework for analyzing drug resistance and advancing personalized medicine in BC. By generating PDOs that faithfully preserve the genomic, transcriptomic, and phenotypic heterogeneity of the original tumors, the study demonstrates that PDOs retain not only receptor status and clonal diversity but also the intrinsic resistance traits present in the patient’s disease. Organoids derived from clinically resistant lesions maintain reduced drug sensitivity in vitro, enabling the controlled study of resistance-associated features, such as altered cellular state composition, compromised apoptotic priming, and microenvironment-independent survival programs. These models allow for systematic testing of alternative therapeutic strategies, including molecular pathway-targeted combinations designed to overcome resistance. When transplanted into mouse hosts, resistant PDOs replicate patient-specific growth kinetics and metastatic behavior, providing a level of in vivo validation that reinforces their translational relevance. It is important to note that the concordance between organoid-based drug responses and murine tumor behavior highlights that resistance mechanisms captured in vitro, such as persistent HER2 signaling, endocrine escape, or enrichment of stem cell subpopulations, remain operational in vivo. This dual-platform approach advances precision oncology by enabling personalized drug screening, early identification of patients who do not respond to treatment, and the rational selection of therapies capable of bypassing or suppressing resistant clones [6]. Taken together, these results suggest that imaging-based characterization of resistance should go beyond the intrinsic mechanisms of cancer cells to include dynamic changes within the surrounding microenvironment. The integration of imaging data derived from organoids with longitudinal in vivo imaging therefore provides a more comprehensive framework for studying therapeutic escape and designing new intervention strategies.

Multiscale imaging approaches have significantly advanced the study of metastatic dissemination and therapeutic resistance in preclinical BC models. Duman and colleagues present a brief review that provides an overview of the most widely used in vitro models for studying BC cell migration, highlighting their respective advantages, limitations, and future prospects [87]. Microfluidic tumor models, vascularized organoids, and patient-derived platforms provide complementary systems for investigating metastatic dissemination and TME interactions across different biological scales [88]. Combined with advanced imaging in murine models, these approaches enable longitudinal characterization of metastatic progression and organ-specific colonization, strengthening the translational relevance of preclinical breast cancer research [89].

In a recent study, Zhao et al. developed a microfluidics-based platform for the bottom-up engineering of vascularized 3D tumor models, designed to better recapitulate the structural and functional complexity of the TME. Using droplet microfluidics and bioengineered hydrogel matrices, the authors generated multicellular tumor constructs incorporating cancer cells, endothelial cells, and stromal components, enabling the formation of perfusable microvascular networks within the 3D tissue. The platform allowed precise control over cellular composition, spatial organization, and nutrient delivery, thereby reproducing key physiological features that are often absent in conventional spheroid and organoid cultures. High-resolution FLI and quantitative analysis were used to monitor vascular network formation, tumor growth, cell–cell interactions, and drug penetration within the engineered tissues. Functional validation demonstrated that the presence of vascular structures significantly influenced therapeutic response, drug distribution, and tumor cell viability, highlighting the critical role of the microenvironment in modulating treatment efficacy. Compared with traditional 2D and non-vascularized 3D models, the vascularized microfluidic tumors exhibited more physiologically relevant responses to anticancer agents and provided improved predictive power for preclinical drug evaluation. The study underscores the potential of microfluidic vascularized tumor models as advanced platforms for drug screening, mechanistic studies of tumor–stroma interactions, and the development of personalized therapeutic strategies [90]. Prince and colleagues have developed a microfluidic platform containing arrays of patient-derived breast tumor spheroids for high-throughput drug screening. Using longitudinal FLI, the authors quantified spheroid viability and treatment responses, revealing significant heterogeneity among patients and differential drug sensitivities. The study underscores the importance of imaging-based 3D microfluidic tumor models for identifying mechanisms of drug resistance and for supporting the selection of personalized treatments in precision oncology [91]. Pradhan et al. have developed a physiologically relevant in vitro system that replicates the key characteristics of the vascularized breast TME. By integrating metastatic and non-metastatic BC cells with stromal fibroblasts within a PEG-fibrinogen hydrogel and coupling this compartment to a perfused, lumenized endothelial network, the model generates spatially heterogeneous regions in terms of perfusion, nutrient availability, and stromal interaction that faithfully mirror the in vivo tumor architecture. This configuration enables the quantitative assessment of drug penetration, endothelial toxicity, and tumor-specific cytotoxicity under controlled flow conditions, revealing differential therapeutic responses determined by microvascular organization and interstitial transport. This platform, which has demonstrated the ability to model vascular permeability, perfusion gradients, and stroma–tumor interaction, provides a mechanistic bridge between conventional organoid assays and perfusion- and permeability-based endpoints detected by modalities such as DCE-MRI and CEUS in murine models [92]. Although these systems substantially improve the physiological relevance of conventional in vitro models, significant challenges remain regarding their scalability, reproducibility, and standardization across different laboratories. Addressing these issues will be essential before such platforms can be systematically integrated into preclinical drug development processes.

Khan et al. introduced a high-resolution positron emission microscopy platform (oPEM) that enables PET-like functional imaging in PDOs using clinical radiotracers. By directly comparing oPEM results with the PET scans of the corresponding patients, the study establishes a rare bidirectional link between ex vivo organoid metabolism and in vivo functional imaging, demonstrating that microscale FDG uptake patterns in PDOs recapitulate the metabolic behavior of the original tumors. Notably, Khan et al. did not employ any animal models; instead, they performed high-resolution positron emission microscopy directly on PDOs and compared these microscale metabolic signatures with the PET scans of the corresponding patients. This animal-free design establishes a unique direct link between PDO metabolism and in vivo clinical imaging [93]. Mazzucchelli et al. employed exclusively in vitro imaging modalities, including confocal microscopy, scanning electron microscopy, transmission electron microscopy, and immunofluorescence, to characterize morphological evolution, lineage composition, and ultrastructural changes in PDOs before and after therapy. Although the study does not incorporate in vivo imaging, its high-resolution morphological endpoints provide a complementary layer to functional readouts obtained in murine models. In the context of a PDO → PDX → imaging pipeline, such detailed in vitro phenotyping helps identify early cellular adaptations and resistance-associated features that can later be validated through whole-organism imaging modalities, thereby strengthening the translational continuity between organoid-based assays and in vivo therapeutic response [94]. Overall, these studies demonstrate how complementary imaging technologies can analyze the biology of breast cancer across multiple spatial scales, from subcellular architecture to whole-organism physiology. Rather than replacing animal models, advanced 3D platforms are increasingly providing mechanistic insights that can be validated through longitudinal in vivo imaging, supporting a multiscale framework for translational BC research.

### 4.3. AI-Driven Imaging

Artificial intelligence (AI)-based imaging is playing an increasingly central role in multiscale cancer modeling, enhancing both in vitro and in vivo analyses through the automation of segmentation, feature extraction, and radiomic profiling [95]. Machine learning (ML) and deep learning (DL) approaches reduce operator-dependent variability and enable the identification of complex imaging patterns associated with tumor phenotype, therapeutic response, and disease progression. In organoid systems, AI-assisted image analysis enables quantitative assessment of morphology, growth dynamics, cellular organization, and phenotypic heterogeneity, supporting high-throughput drug screening and objective evaluation of treatment-induced changes. Image-based profiling approaches combined with computational analysis have demonstrated the ability to identify morphological signatures associated with drug response and cellular states in BC models, highlighting the potential of AI-driven imaging pipelines for biomarker discovery [96]. Similarly, DL-based approaches for automated organoid segmentation, detection, and longitudinal tracking have improved the reproducibility and throughput of quantitative measurements in complex 3D cancer models, enabling more robust assessment of organoid growth, morphology, and treatment response [97,98]. In murine imaging, AI-based methods are increasingly applied to analyze multimodal datasets, including MRI, PET, CT, CEUS, and OI, facilitating automated tumor segmentation, volumetric quantification, and longitudinal monitoring. These approaches enable the identification of spatial and temporal patterns associated with therapeutic response, metastatic potential, and emerging resistance [95]. Beyond conventional image analysis, radiomics has emerged as a powerful strategy for extracting high-dimensional quantitative imaging features that capture tumor heterogeneity beyond visual interpretation. Radiomic signatures derived from multimodal imaging datasets can characterize the morphological, metabolic, and functional properties of tumors, providing quantitative biomarkers for treatment response prediction and patient stratification. In BC, radiomics-based approaches have been investigated for molecular subtype classification, prediction of therapeutic response, and identification of prognostic biomarkers [99]. In addition to feature extraction, multimodal AI frameworks integrate imaging with genomic, transcriptomic, proteomic, and other multi-omic datasets to generate predictive signatures that bridge experimental models and clinical applications. Radiogenomic approaches have further expanded this concept by linking imaging phenotypes with molecular pathways, tumor subtypes, and therapeutic vulnerabilities, thereby supporting biomarker discovery and patient stratification strategies [100]. Recent advances in radiogenomics and multi-omics integration have highlighted the potential of combining imaging-derived features with molecular information to identify biologically relevant imaging biomarkers and improve precision oncology approaches [101].

**Figure 3:**
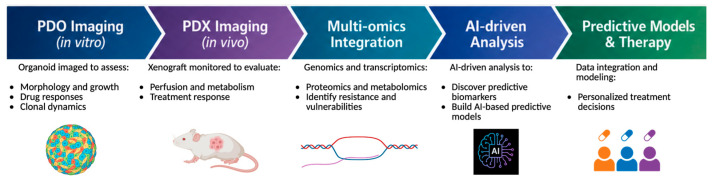
Integrated pipeline for imaging, multi-omics, AI and personalized therapy. Schematic representation of the translational workflow combining PDO imaging (in vitro), PDX imaging (in vivo), multi-omics integration and AI-driven analysis to generate predictive models for personalized cancer therapy (created with BioRender.com).

In this context, patient-derived avatars, including PDOs, PDXs, and organoid-on-a-chip, provide a translational continuum from cellular physiology to whole-organism biology when combined with longitudinal imaging. In vitro avatars enable rapid image-based assessment of drug combinations, clonal dynamics, and treatment responses, whereas in vivo avatars validate these findings within the context of stromal interactions, immune components, and pharmacokinetic constraints. Longitudinal OI in PDOs together with functional MRI or PET in PDX models can reveal resistant subclones, compensatory signaling pathways, and microenvironmental remodeling associated with disease progression and recurrence. When imaging-based predictions generated by these avatars are prospectively validated in complementary murine models, they can substantially strengthen their translational value [102]. Integrating imaging with multi-omic approaches further enhances the interpretation of spatially resolved phenotypes by linking imaging features with molecular programs and biological mechanisms [103]. Taken together, these advances make AI-driven imaging a fundamental integrative platform in the field of multiscale cancer modeling, linking phenotypic, molecular, and functional information to improve biomarker identification, treatment prediction, and the clinical translation of precision oncology approaches (see Figure 3).

## 5. Discussion

The growing complexity of BC biology requires experimental models capable of capturing tumor heterogeneity, interactions with the microenvironment, metastatic spread, and response to treatment across multiple biological scales. As highlighted throughout this review, no single preclinical model is sufficient to replicate all aspects of the human disease. Instead, the integration of complementary platforms, including organoids, CAM assays, mouse models, and advanced imaging technologies, provides a more comprehensive framework for studying BC progression and its therapeutic vulnerability.

Three-dimensional organoid systems have significantly improved the physiological relevance of in vitro studies by preserving the fundamental histological and molecular characteristics of the original tumors. Their compatibility with high-content imaging approaches allows for detailed characterization of cellular architecture, invasion dynamics, and drug responses. However, the absence of fully functional vascular, immune, and stromal compartments limits their ability to reproduce the complexity of the tumor ecosystem. Recent developments in co-culture systems, organ-on-chip technologies, and vascularized spheroid models are beginning to overcome these limitations, providing intermediate platforms that bridge the gap between conventional organoid cultures and animal models.

The CE model represents a further translational step between in vitro systems and mammalian models. Its highly vascularized environment, rapid experimental turnaround times, and reduced ethical impact make it particularly attractive for preliminary therapeutic screening and studies of tumor–host interactions. Furthermore, the recent implementation of imaging techniques such as HFUS and MRI demonstrates the CAM platform’s potential for longitudinal and noninvasive investigations. Although it cannot completely replace mouse models, the CAM assay can contribute substantially to reducing animal use while preserving biological complexity.

Mouse models remain indispensable for studying systemic tumor progression, metastatic colonization, and response to treatment in a physiologically integrated environment [104]. GEMM models offer unique opportunities to study tumor onset and evolution in the presence of an intact immune system, while CDX and PDX models allow for the evaluation of subtype-specific therapeutic strategies and patient-derived biological variability [105]. However, each model has inherent limitations, including a limited representation of human immune responses, variable metastatic behavior, and difficulties in reproducing the full spectrum of tumor heterogeneity. Consequently, the combination of multiple modeling systems is increasingly recognized as the most effective strategy for improving translational relevance in the context of precision oncology.

Advanced imaging technologies provide complementary molecular, cellular, anatomical, and functional information across all experimental platforms, enabling longitudinal assessment of tumor progression and therapeutic response. The growing adoption of multimodal imaging approaches enables the longitudinal assessment of tumor growth, vascular remodeling, metabolism, immune interactions, and therapeutic response within the same biological system. It is important to note that many imaging biomarkers identified in preclinical studies are directly transferable to clinical practice, strengthening the bridge between laboratory research and personalized medicine. A particularly promising area involves the integration of imaging with multi-omic analyses and computational tools based on AI. Quantitative biomarkers derived from imaging can be combined with genomic, transcriptomic, proteomic, and metabolomic datasets to generate predictive models of disease progression and therapeutic response or resistance. ML algorithms are increasingly capable of extracting biologically meaningful patterns from complex imaging datasets, facilitating automated phenotyping, therapeutic stratification, and biomarker discovery [106]. Such approaches could ultimately support the development of patient-specific therapeutic strategies and improve the predictive value of preclinical studies.

### Challenges and Future Perspectives

Despite this progress, several challenges remain. The standardization of imaging protocols, the harmonization of analytical pipelines, and the validation of biomarkers across different experimental platforms are still needed to improve reproducibility and facilitate clinical translation. Furthermore, the generation of large, well-annotated datasets that integrate imaging and molecular information will be essential to fully harness the potential of artificial intelligence and systems biology approaches.

Addressing these challenges will require the development of truly multiscale imaging pipelines that rely on the rational integration of complementary preclinical models rather than on the identification of a single optimal experimental system [107]. PDOs provide highly informative platforms for high-throughput drug screening and mechanistic studies, whereas vascularized organoids and organ-on-chip technologies more closely reproduce key features of the TME that are absent in conventional 3D cultures. Combined with intermediate platforms such as the CE CAM model for rapid evaluation of tumor vascularization, angiogenesis, and early therapeutic responses, these systems can guide the selection of candidate therapies before validation in orthotopic or PDX murine models, where systemic physiology, immune interactions, metastatic dissemination, and longitudinal treatment efficacy can be comprehensively assessed. Such sequential multiscale workflows are likely to provide the greatest predictive value for preclinical therapeutic evaluation by capitalizing on the complementary strengths of each experimental platform.

Within this integrated framework, multimodal imaging technologies will play a central role by connecting biological information across cellular, tissue, and whole-organism levels. Rather than functioning as isolated techniques, OI, HFUS, MRI, PET/CT, and IVM provide complementary molecular, functional, anatomical, and cellular information that can be integrated throughout the preclinical pipeline. Among the imaging biomarkers currently showing the greatest translational potential are DWI-MRI-derived apparent diffusion coefficient (ADC), dynamic contrast-enhanced MRI parameters, FDG-PET metabolic indices, and receptor-targeted PET tracers, all of which have demonstrated encouraging validation across both preclinical and clinical BC studies [62,108,109]. Nevertheless, broader clinical implementation will require standardized multicenter validation to ensure reproducibility across imaging platforms.

However, significant practical barriers still limit the routine implementation of integrated multiscale workflows. The successful integration of multimodal imaging requires access to highly specialized imaging infrastructures, whose acquisition, maintenance, and operation entail substantial financial investments that often exceed the resources of individual laboratories. Consequently, collaborative imaging facilities and shared technological infrastructures will be increasingly important to facilitate access to advanced imaging technologies and promote standardized experimental workflows. In parallel, regulatory and ethical considerations remain central to the design of multiscale preclinical studies. Although advanced in vitro platforms, including PDOs and organ-on-chip systems, contribute to the implementation of the 3Rs principles by reducing and refining animal experimentation, in vivo validation remains essential for investigating complex biological processes such as immune responses, pharmacokinetics, tumor evolution, and metastatic dissemination.

An additional challenge concerns the integration and harmonization of the heterogeneous datasets generated by multimodal imaging. Variability in acquisition parameters, scanner performance, image reconstruction, segmentation procedures, and quantitative analysis methods continues to limit reproducibility and cross-study comparisons. Addressing this bottleneck will require standardized acquisition protocols, validated quantitative imaging biomarkers, and harmonized analytical pipelines. Ongoing international initiatives, including the National Electrical Manufacturers Association (NEMA) performance standards for molecular imaging systems and the Quantitative Imaging Biomarkers Alliance (QIBA), together with FAIR (Findable, Accessible, Interoperable, and Reusable) data principles and interoperable computational workflows, represent important steps toward improving multicenter reproducibility and accelerating the clinical translation of imaging biomarkers.

Overall, the future of BC research lies in the convergence of advanced experimental models, multimodal imaging, and computational analysis. The integration of complementary biological models, quantitative imaging, and interoperable computational frameworks is expected to improve the predictive value of preclinical studies, accelerate biomarker discovery, and facilitate the translation of imaging-guided precision oncology into clinical practice.

## Figures and Tables

**Table 1:** Comparative overview of preclinical BC models.

Model	Main Applications	Strengths	Limitations	Imaging Modalities Best Suited
**3D Organoids**	Drug screening, tumor biology, personalized medicine	Human-derived, preserves heterogeneity, high-throughput	Lack systemic interactions	Fluorescence, confocal, multiphoton, FLIM
**Vascularized 3D Models**	Angiogenesis, immune interactions	More physiological microenvironment	Technically demanding	Fluorescence, confocal, live-cell imaging
**Chicken Embryo (CAM)**	Angiogenesis, invasion, rapid drug testing	Low cost, rapid, 3Rs, intermediate model	Short experimental window, immature immune system	Optical imaging, HFUS, MRI
**GEMMs**	Tumor initiation, progression, immune interactions	Native TME, spontaneous tumor evolution	Time-consuming, expensive	MRI, PET, HFUS, IVM
**CDXs**	Proof-of-concept, imaging probe validation	Reproducible, easy to establish	Limited heterogeneity	BLI, PET, MRI, CT
**PDXs**	Precision oncology, biomarker validation	Preserve patient heterogeneity	Cost, long establishment, immunodeficient host	MRI, PET, CT, Optical imaging

**Table 2:** Comparative overview of imaging modalities employed in preclinical BC models, highlighting their principal applications and translational potential. Qualitative scoring: Imaging modalities and practical characteristics are qualitatively rated as Low, Moderate, High, or Very High, while longitudinal monitoring suitability is rated as High or Excellent. Ratings represent general comparisons across imaging modalities used in preclinical BC research. Abbreviations: BLI, bioluminescence imaging; FLI, fluorescence imaging; OI, optical imaging; HFUS, high-frequency ultrasound; MRI, magnetic resonance imaging; PET/CT, positron emission tomography/computed tomography; PET/MRI, positron emission tomography/magnetic resonance imaging; OI/CT, optical imaging/computed tomography; CEUS, contrast-enhanced ultrasound; DWI, diffusion-weighted imaging; DCE-MRI, dynamic contrast-enhanced magnetic resonance imaging; NIR, near-infrared; TME, tumor microenvironment.

Imaging Modality	Primary Applications	Spatial Resolution	Sensitivity	Relative Cost	Longitudinal Monitoring	Translational Potential	Main Advantages	Main Limitations
**OI** **—** **(BLI)**	Tumor burden assessment; Metastatic dissemination; Therapy response; Cell tracking	Low	Very High	Low	Excellent	Low	Extremely sensitive; Rapid; Inexpensive; Non-invasive	Poor anatomical localization; Limited tissue penetration; Requires luciferase-expressing cells
**OI** **—** **(FLI)**	Molecular imaging; Image-guided surgery; Lymphatic mapping; Visualization of TME interactions	Moderate–High	High	Low–Moderate	Excellent	Moderate	High spatial resolution; Targeted fluorescent probes; NIR imaging	Tissue autofluorescence; Limited penetration depth; Reduced quantitative accuracy in deep tissues
**HFUS**	Tumor volume measurement; Vascularization; Perfusion imaging (Doppler, CEUS); Elastography	Very High	Moderate	Moderate	Excellent	High	Real-time imaging; High spatial resolution; No ionizing radiation; Relatively inexpensive; Functional vascular assessment	Operator-dependent; Limited penetration depth; Limited molecular information
**MRI**	Anatomical characterization; Tumor heterogeneity; TME analysis; DWI; DCE-MRI; Therapy monitoring	Very High	Moderate	High	Excellent	Very High	Excellent soft-tissue contrast; Multiparametric imaging; No ionizing radiation	High cost; Long acquisition times; Lower molecular sensitivity; Specialized instrumentation
**PET/CT**	Metabolic imaging; Proliferation; Receptor expression; Whole-body disease assessment; Therapy response	Moderate	Very High	High	High	Very High	Highly sensitive; Quantitative whole-body imaging; Early detection of biological changes	Limited spatial resolution; Ionizing radiation, Requires radiotracers
**Hybrid imaging** **(PET/MRI, PET/CT, OI/CT)**	Integrated anatomical, functional and molecular characterization; Imaging biomarker development; Comprehensive therapy assessment	High	Very High	Very High	High	Very High	Combines complementary information from multiple modalities; Improves lesion characterization; Strong translational relevance	High cost; Technical complexity; Limited availability

## Data Availability

No new data were created or analyzed in this study. Data sharing is not applicable to this article.
